# Phytoextraction of cadmium-contaminated soil and potential of regenerated tobacco biomass for recovery of cadmium

**DOI:** 10.1038/s41598-017-05834-8

**Published:** 2017-08-03

**Authors:** Yang Yang, Yichen Ge, Hongyuan Zeng, Xihong Zhou, Liang Peng, Qingru Zeng

**Affiliations:** 1 College of Bioscience and Biotechnology, Hunan Agricultural University, Changsha, 410128 China; 2 College of Resources and Environment, Hunan Agricultural University, Changsha, 410128 China; 3 Hunan Provincial Key Laboratory of Rural Ecosystem Health in Dongting Lake Area, Changsha, 410128 China

## Abstract

The aim of this study was to estimate the influence of regenerated tobacco on the extraction of Cd from two acidic soils as well as to address the problem of how to deal with contaminated leaves following phytoextraction. Results showed that a coppicing tobacco led to a decline in Cd concentration in regenerated leaves and stalks when plants were grown in pots, but increased concentrations in regenerated lower and middle leaves when plants were grown under field conditions. The highest recorded bioconcentration factors in Chaling and Guanxi soil were 37.53 and 19.21 in lower leaves in the field, respectively. Total Cd extraction efficiency in practice (9.43% for Chaling soil and 6.24% for Guanxi soil) under field conditions confirmed our theoretical calculations (10.0% for Chaling soil and 6.73% for Guanxi soil). Use of a 0.5% hydrochloric acid(HCl) solution was sufficient to reduce Cd (98.4%) in tobacco leaves to permissible levels as required by the Hygienic Standard for Feeds in China (≤0.5 mg kg^−1^). Regenerated tobacco has the potential to allow cultivation of Cd contaminated farmland to produce animal feed, assist in lowering total Cd content of soil, and allow income generation for farmers.

## Introduction

Contamination of agricultural soil is becoming an increasingly serious problem because of long-term exposure to pollutants in irrigation water, sludge, fertilizers, and pesticides^1^. The non-essential element cadmium (Cd) is one of the most dangerous and potentially toxic metals present in the biosphere, highly mobile in soils, water, and the atmosphere, and causing damage even at low concentrations^2–4^. Pollution by Cd is especially significant as contaminated soils are used for agriculture; this metal is easily absorbed from soils into crops, inhibiting plant growth and nutrient uptake. The increasing presence of Cd in the environment has led to considerable concerns over the last decade^5, 6^; contamination also represents a serious risk to human health as Cd is easily transferred within the food chain^7^.

It is an urgent requirement to either clean Cd from contaminated soil or control its uptake by crops, as these can be consumed by animals and humans. A number of technological developments aimed at achieving this, including soil washing, excavation, and land filling, are wasteful and expensive, and often destroy soil structure and fertility^7^. One promising alternative approach is phytoextraction which is a remediation technique that uses the ability of the plant to uptake heavy metals from soil^8^. Although this technique has a lower cost and is less harmful to the soil environment, a longer time is required to reduce soil heavy metal concentrations to levels that conform to environment standards^9, 10^. In reality, because farmland area is being reduced in some densely populated countries like China as population levels increase and cities expand, areas of contaminated soil are already exploited for agriculture. It is thought that 19.4% of farmland in China is currently contaminated^11^, which leads to serious economic losses for both farmers and local government. Therefore, if generated biomass can be switched to an alternative revenue stream that augments the livelihood of farmers, then the time needed to decontaminate soil may become less critical and slower phytoextraction projects based on the gradual attenuation of heavy metals rather than short-term forced removal may be possible^12, 13^.

Tobacco (*Nicotiana tabacum* L.) is a commercial crop that fits the criteria for phytoextraction. It is fast growing, easily-harvested, generates a high biomass yield, and can accumulate high levels of Cd in tissues even under conditions of low exposure. Cd levels in most plant tissues decrease in the order: root > leaf > fruit > seed^14^; however, in tobacco, concentrations are higher in leaves than in the other parts of the plant^15^. Various studies have shown that tobacco has enhanced capabilities to uptake Cd from soil and to accumulate it in high volumes in leaves^16, 17^. Indeed, the results of pot and hydroponic experiments have shown that, compared to other crops, tobacco is able to accumulate relatively higher concentrations of Cd. One greenhouse experiment showed, for example, that Cd concentrations in tobacco leaves reached 86.9 mg kg^−1^ and 271.5 mg kg^−1^, respectively, when nutrient solutions of 0.25 mg L^−1^ and 1.0 mg L^−1^ cadmium chloride (CdCl_2_) were applied^18^. Tobacco cultivated hydroponically and subjected to a 1.5 mg L^−1^ CdCl_2_ solution will grow normally without visible symptoms of toxicity while at the same time accumulating Cd concentrations of up to 226 mg kg^−1^ in its lower leaves.

The majority of recent field and experimental studies have focused on the effects of Cd on the growth, development, physiology, and biochemical properties of tobacco, as well as on the differences between the various cultivars in Cd uptake and tolerance^12, 19, 20^. Little comprehensive information is currently available regarding the accumulation of Cd in regenerated tobacco and the residual products of phytoextraction which need to be disposed of appropriately. Thus, to fully utilize tobacco for phytoremediation and to enhance the economic value of this crop, the aims of this study were: (1) to determine the absorption and enrichment of Cd in tobacco before and after regeneration in two different contaminated soils on the basis of pot and field experiments; (2) to investigate whether the phytoextraction efficiency of Cd from contaminated soil can be enhanced by regenerated tobacco; and (3) to assess the possibility of using HCl as an extractant for the removal of Cd from contaminated tobacco leaves and to evaluate potential uses of Cd depleted leaves as animal feed.

## Results

### Cadmium accumulation in tobacco

**Figure 1:**
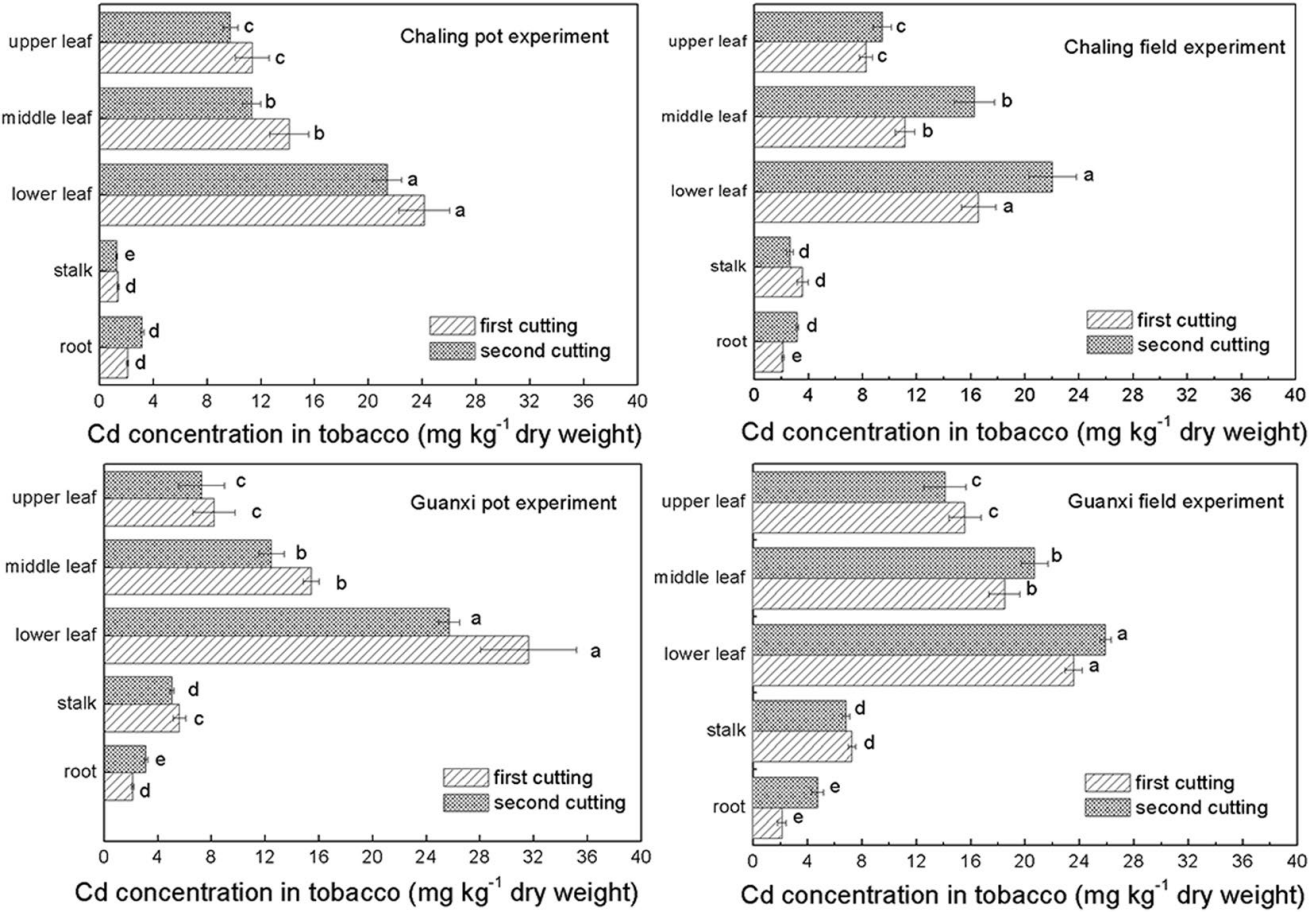
Cd concentrations in tobacco grown in pot and field experiments. Values are means ± SE for nine replicates, while different letters indicate significant differences between root, stalk, lower leaf, middle leaf, and upper leaf of tobacco according to Duncan’s multiple range test at *P* ≤ 0.05.

Results of Cd concentrations and BCF (bioconcentration factors) values for tobacco at two cutting times in our pot experiments are presented in Fig. 1 and Table 1. These data show considerable differences in the distribution of Cd in tobacco tissues (*P* ≤ 0.05). Cd levels in spring growth and regrown tobacco occur in the order, lower leaves > middle leaves > upper leaves > root > stalk (or > stalk > root). Following the first cutting of plants grown in Chaling soil, the proportions of Cd in upper, middle, and lower leaves were 11.33 mg kg^−1^, 14.08 mg kg^−1^, and 24.13 mg kg^−1^, respectively, while BCF values were high, up to 19.31, 23.99, and 41.11, respectively. In Guanxi pot experiments, total Cd was 1.34 mg kg^−1^ in soil, while the highest recorded concentration was 31.59 mg kg^−1^ in lower leaves following the first cut, corresponding with a BCF value of 23.45. With the exception of Cd concentrations in upper leaves, the proportion of this element in other parts of tobacco plants grown in Guanxi soil were higher than in plants grown in the Chaling soil. Results also show that cutting treatment had an effect on pot experiments using both soil types, and that the second cut resulted in a lower Cd concentration in tobacco leaves and stalks.

**Table 1:** BCF values for tobacco grown in pot and field experiments.

Soil	Cutting times	Upper leaf	Middle leaf	Lower leaf	Stalk	Root
Chaling	Pot-1st	19.31 ± 2.16^a^	23.99 ± 2.48^b^	41.11 ± 3.22^a^	2.32 ± 0.14^c^	3.51 ± 0.16^b^
	Pot-2nd	16.56 ± 0.91^b^	19.25 ± 1.16^c^	36.41 ± 1.82^a^	5.38 ± 0.19^c^	2.14 ± 0.10^a^
	Field-1st	14.04 ± 0.83^b^	18.96 ± 1.26^c^	28.23 ± 2.16^b^	6.05 ± 0.70^a^	3.60 ± 0.15^b^
	Field-2nd	16.09 ± 1.18^b^	27.71 ± 2.54^a^	37.53 ± 2.99^a^	4.51 ± 0.41^b^	5.37 ± 0.18^a^
Guanxi	Pot-1st	6.07 ± 1.17^b^	11.44 ± 0.44^c^	23.45 ± 2.64^a^	4.16 ± 0.34^b^	1.58 ± 0.06^c^
	Pot-2nd	5.38 ± 1.26^b^	9.24 ± 0.70^d^	19.07 ± 0.58^a^	3.75 ± 0.12^b^	2.31 ± 0.10^b^
	Field-1st	11.55 ± 0.89^a^	13.71 ± 0.84^b^	17.48 ± 0.46^a^	5.38 ± 0.19^a^	1.55 ± 0.21^c^
	Field-2nd	10.46 ± 1.14^a^	15.34 ± 0.74^a^	19.21 ± 0.30^a^	5.06 ± 0.21^a^	3.51 ± 0.31^a^

Note: Standard deviation is represented here as ±SE (n = 3); columns with different letters indicate a significant difference at the *P* ≤ 0.05 level on the basis of Duncan’s multiple range test.

Results show that Cd accumulation in tobacco grown under field conditions in the same soil subsequent to first cutting (Fig. 1 and Table 1) was significantly lower than under pot conditions (Fig. 1). Cd concentration following the first cut also varied between tobacco leaves, from 8.24 mg kg^−1^ in upper leaves to 16.57 mg kg^−1^ in lower leaves in the Chaling soil field, and from 15.56 mg kg^−1^ in upper leaves to 23.55 mg kg^−1^ in lower leaves in the Guanxi soil field. Following the second cutting in the field experiment, regenerated tobacco leaves (with the exception of upper leaves in the Guanxi soil field) showed a marked difference compared to those in the pot experiment; concentrations of Cd in lower and middle regenerated leaves were elevated to 22.03 mg kg^−1^ and 16.27 mg kg^−1^, respectively, in the Chaling soil field, and elevated to 25.88 mg kg^−1^ and 20.67 mg kg^−1^, respectively, in the Guanxi soil field. In addition, BCF values of regenerated tobacco plants grown under field conditions were also enhanced and higher than in pot experiments (Table 1).

### Tobacco biomass production and heavy metal uptake

None of the tobacco plants considered in this study exhibited symptoms of deficiency and yields recorded were within the normal range for plants in the local area. Variation in tobacco yields between the two cutting times in the field experiment were generally more obvious than those seen in pot experiments (Table 2, *P* ≤ 0.05), potentially due to the more complex field environment. Average total leaves weights measured in pot experiments ranged between 194.93 g plant^−1^ and 197.40 g plant^−1^, with upper and middle leaves accounting for over 70% of this mass. Stalk weights ranged between 62.16 g plant^−1^ and 63.30 g plant^−1^, while under field conditions, the total biomass of tobacco leaves and stalks were in the ranges 4.24 ton ha^−1^ to 5.03 ton ha^−1^ and 1.41 ton ha^−1^ to 1.48 ton ha^−1^, respectively.

**Table 2:** Biomass of tobacco leaves and stalks.

Pot experiment	Cutting time	Upper leaf	Middle leaf	Lower leaf	Total leaves	Stalk
		g plant^−1^				
Chaling	1st	72.32 ± 0.60^a^	73.41 ± 1.31^a^	49.20 ± 1.56^a^	194.93 ± 0.92^b^	63.30 ± 0.90^a^
	2nd	73.15 ± 0.76^a^	74.25 ± 1.36^a^	50.00 ± 0.71^a^	197.40 ± 0.53^a^	63.16 ± 1.05^a^
Guanxi	1st	73.55 ± 1.06^a^	73.98 ± 1.00^a^	49.15 ± 0.57^a^	196.67 ± 1.87^ab^	62.67 ± 1.32^a^
	2nd	73.04 ± 0.88^a^	73.56 ±1.23^a^	49.87 ± 2.22^a^	196.47 ± 0.97^ab^	62.53 ± 0.55^a^
**Field experiment**	**Cutting time**	**Upper leaf**	**Middle leaf**	**Lower leaf**	**Total leaves**	**Stalk**
		**t ha** ^**-1**^				
Chaling	1st	1.86 ± 0.02^a^	1.86 ± 0.02^a^	1.31 ± 0.03^a^	5.03 ± 0.03^a^	1.47 ± 0.020^a^
	2nd	1.65 ± 0.02^b^	1.64 ± 0.03^b^	1.13 ± 0.03^b^	4.42 ± 0.08^b^	1.42 ± 0.024 ^b^
Guanxi	1st	1.85 ± 0.04^a^	1.85 ± 0.02^a^	1.28 ± 0.02^b^	4.98 ± 0.06^a^	1.48 ± 0.017^a^
	2nd	1.65 ± 0.02^b^	1.49 ± 0.01^c^	1.12 ± 0.01^b^	4.24 ± 0.01^c^	1.41 ± 0.018 ^b^

Note: Standard deviation is represented as ±SE (n = 3); columns with different letters indicate a significant difference at the *P* ≤ 0.05 level on the basis of Duncan’s multiple range test.

Total Cd uptake by tobacco plants that were cut twice is shown in Table 3. These results show that the total theoretical extraction of Cd from soil by these plants in pot experiments averaged 5.82 mg pot^−1^ and 6.69 mg pot^−1^ for the Chaling and Guanxi soils, respectively. Theoretical percentages of extracted Cd correspond with these results, up to 66.16% and 33.09% for the Chaling and Guanxi soils, respectively. Based on field experiments, total theoretical extraction of Cd by tobacco was 132.06 g ha^−1^ and 203.91 g ha^−1^ for Chaling and Guanxi soils, respectively, while theoretical extraction efficiencies of Cd by field tobacco were 10.00% and 6.73% for Chaling and Guanxi soils, respectively, significantly lower than pot experiments.

**Table 3:** Cd uptake and phytoextraction efficiency of tobacco grown in different soils after two cuts.

Soil	Cutting time	Cd uptake by tobacco	
		Pot experiment mg pot^−1^	Field experiment g ha^−1^
Chaling	1st	3.13 ± 0.02^d^	61.15 ± 1.77 ^f^
	2nd	2.70 ± 0.04 ^f^	70.91 ± 1.41^e^
	Total	5.82 ± 0.02^b^	132.06 ± 3.19^b^
Guanxi	1st	3.64 ± 0.03^c^	107.65 ± 1.28^c^
	2nd	1.88 ± 0.04^e^	96.26 ± 0.24^d^
	Total	5.52 ± 0.07^a^	203.91 ± 1.73^a^
**Soil**	**Cutting time**	**Phytoextraction efficiency %**	
		**Pot experiment**	**Field experiment**
Chaling	1st	35.51 ± 0.22^b^	4.63 ± 0.13^d^
	2nd	30.64 ± 0.41^d^	5.37 ± 0.11^c^
	Total	66.16 ± 0.26^a^	10.00 ± 0.25^a^
Guanxi	1st	18.04 ± 0.16^e^	3.55 ± 0.05^e^
	2nd	15.05 ± 0.19 ^f^	3.18 ± 0.01^f^
	Total	33.09 ± 0.35^c^	6.73 ± 0.06^b^

Note: Standard deviation is represented as ±SE (n = 3); columns with different letters indicate a significant difference at the *P* ≤ 0.05 level on the basis of Duncan’s multiple range test.

### Total and available Cd in soil

**Figure 2:**
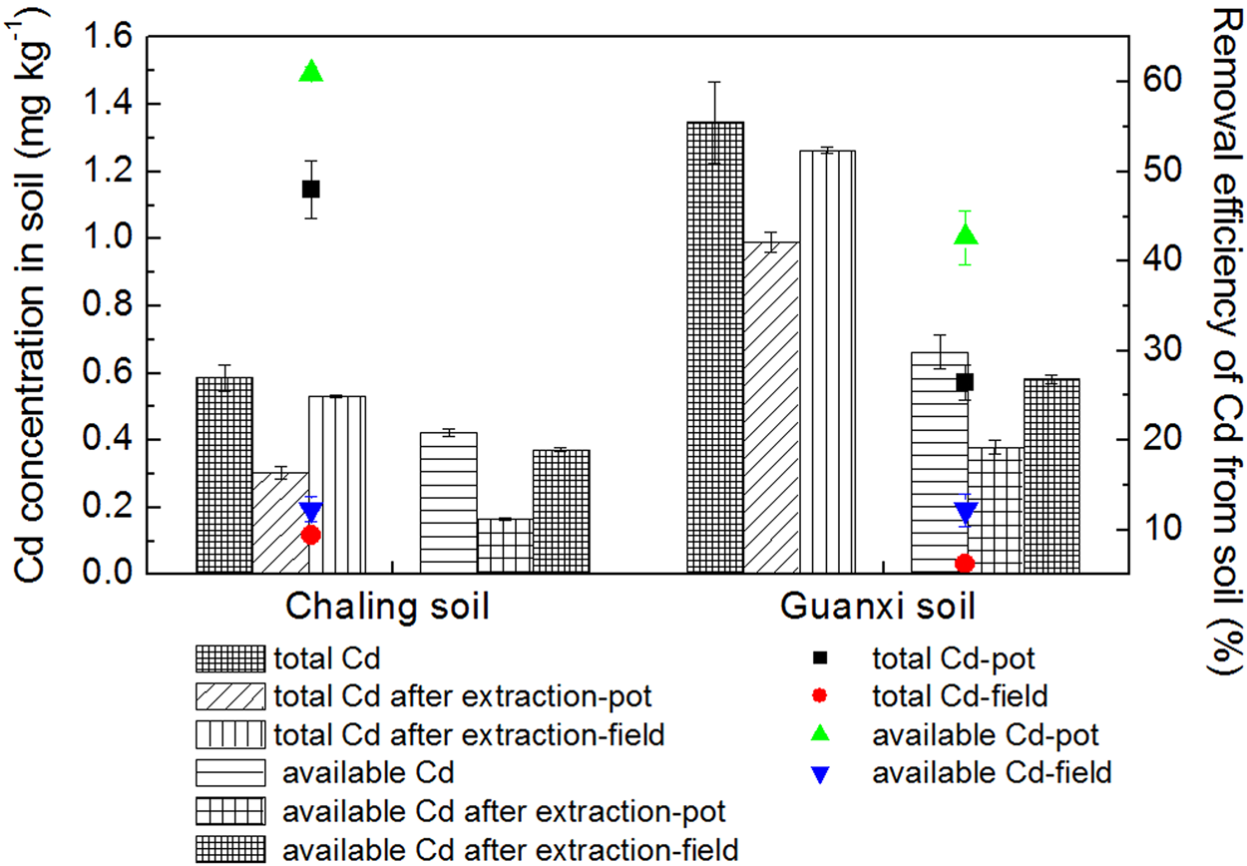
Cd concentrations in soils before and after phytoextraction and Cd removal efficiency by tobacco. Cd concentration in soil (mg kg^−1^) expressed as bar plots with SE of means, while Cd removal efficiency (%) expressed as the symbols ■, ●, ▲ and ▼ represent means with SE.

Results of total and available Cd concentrations in topsoil (0–20 cm depth), before and after phytoextraction following two cuts under pot and field conditions are shown in Fig. 2. In pot experiments, total and available Cd decreased in the Chaling soil from 0.59 mg kg^−1^ and 0.42 mg kg^−1^ to 0.31 mg kg^−1^ and 0.16 mg kg^−1^, respectively, while in the Guanxi soil, these values decreased from 1.35 mg kg^−1^ and 0.66 mg kg^−1^ to 0.99 mg kg^−1^ and 0.38 mg kg^−1^, respectively. The highest measured Cd removal efficiency from soil after two cuts was 48.04% for total Cd and 60.99% for available Cd in the Chaling pot experiment. Results show that both total and available Cd concentrations under field conditions declined less obviously than in pot conditions; removal efficiencies of total and available Cd under field conditions were 9.43% and 12.29% in the Chaling soil, respectively, and 6.24% and 12.21% in the Guanxi soil, respectively.

### Effectiveness of HCl for the removal of Cd from tobacco leaves

**Figure 3:**
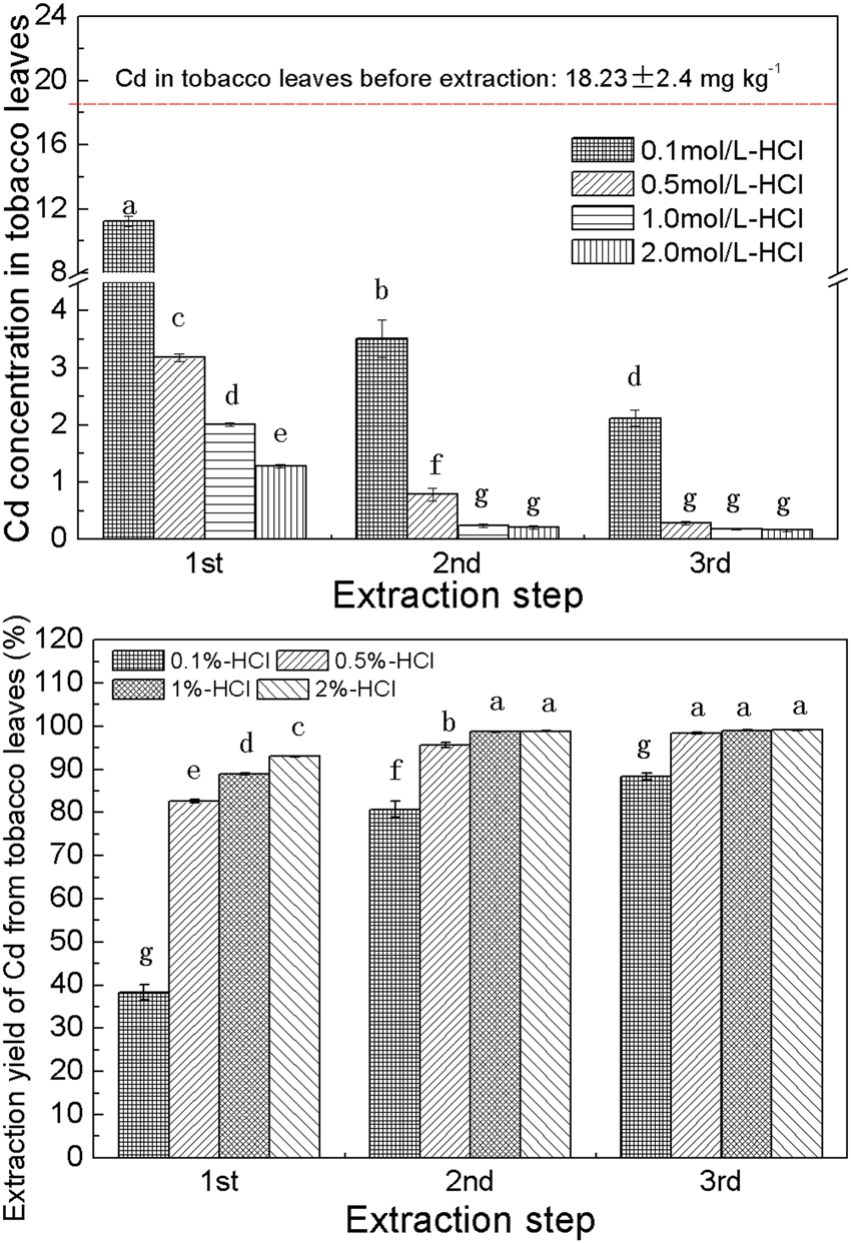
Cd concentrations in tobacco leaves and extraction yield (%) from tobacco leaves after the three step extraction using different concentration of HCl. Bar plots with SE of means, while different letters indicate significant variations between different HCl concentrations and extraction steps on the basis of Duncan’s multiple range test (*P* ≤ 0.05).

The effects of different HCl concentrations on the removal of Cd from mixed tobacco leaves with an initial concentration of 18.23 mg kg^−1^ dry weight are shown in Fig. 3. These results clearly show that HCl has a significant effect on Cd extraction, as this increase gradually along with concentration (*P* ≤ 0.05). In addition, Cd concentration in tobacco leaves decreased markedly, from 18.23 mg kg^−1^ to 11.25 mg kg^−1^, 3.18 mg kg^−1^, 2.01 mg kg^−1^, and 1.28 mg kg^−1^ following first-extractions with 0.1%, 0.5%, 1%, and 2% HCl. On the basis of these results, Cd extraction efficiency was calculated based on the volume of metal removed from tobacco leaves. After first extraction, 2% HCl exhibited a significant high Cd removal efficiency of 92.98%, followed by the subsequent concentrations of 1%, 0.5%, and 0.1%, which had efficiencies of 88.97%, 82.56%, and 38.29%, respectively. In addition, Cd removal efficiency was significantly increased by the addition of successive extraction steps (*P* ≤ 0.05; Fig. 3); Cd concentrations in tobacco leaves decreased to 3.51 mg kg^−1^, 0.79 mg kg^−1^, 0.24 mg kg^−1^, and 0.21 mg kg^−1^, respectively, as HCl concentrations were progressively increased (ranging between 0.1% and 2%). In these cases, up to 80.75%, 95.67%, 98.68%, and 98.83% of total Cd was removed from plants via HCl extraction. Indeed, following three successive extractions, the Cd removal efficiency of tobacco leaves subject to 0.1% HCl (i.e., 88.37% removal efficiency; 2.12 mg kg^−1^ of Cd remained in tobacco leaves) was remarkably lower than that seen at other HCl concentrations. Differences in Cd concentration in tobacco leaves treated with 0.5%, 1%, and 2% HCl following third extraction were not obvious, however, decreasing to 0.29 mg kg^−1^, 0.18 mg kg^−1^, and 0.16 mg kg^−1^, respectively, as up to 98.40%, 99.01%, and 99.12% of Cd were extracted in these cases. These results show that it is not necessary to use high HCl concentrations to extract Cd from tobacco leaves; just a 0.5% HCl solution is sufficient to attain satisfactory removal efficiency, the same as a 2% HCl solution following three successive extractions.

### Changes in nutrients and nicotine in tobacco leaves after extraction

Analytical concentrations of nicotine and nutrients measured in tobacco leaves before and after HCl extraction are presented in Table 4. These data show that variations in Ca, Mg, P, and K in tobacco leaves were significantly larger than differences in Fe and crude protein depending on the different concentrations of HCl used for extraction (Table 4). In particular, in a 2% HCl extraction, concentrations of Ca, Mg, P, and K were dramatically reduced; from 20,951 mg kg^−1^ to 2,896 mg kg^−1^, from 3,780 mg kg^−1^ to 333 mg kg^−1^, from 3,488 mg kg^−1^ to 872 mg kg^−1^, and from 18,745 mg kg^−1^ to 13,697 mg kg^−1^, respectively. In contrast, extraction with 0.1% and 0.5% HCl solutions had little influence on the concentration of nutrient elements (i.e., Ca, Mg, P, and K) compared to extraction with 2% HCl. However, a lower reduction was seen in the content of crude protein and Fe remaining in residual tobacco leaves following extraction with 0.1% HCl compared to a 2% solution; these values decreased from 13.19% to12.17%, and from 221 mg kg^−1^ to 209 mg kg^−1^, similar to the values seen in untreated tobacco leaves. The level of nicotine also decreased markedly, from 2.56% to 0.26%, following extraction with 0.1% HCl. Indeed, almost all the nicotine present in tobacco leaves was removed by three successive extractions using HCl solution concentrations between 0.5% and 2% mixed with 70% ethanol.

**Table 4:** Content of nicotine and nutrients (i.e., crude protein, Ca, Mg, Fe, K, and P) in residual tobacco leaves following extraction.

	Ca	Mg	P	Fe	K	Crude protein%	Nicotine %
	mg kg^−1^						
**Untreated**	20,942 ± 193^a^	3,781 ± 115^a^	3,380 ± 115^a^	234 ± 10^a^	18,817 ± 172^a^	13.93 ± 0.27^a^	2.52 ± 0.04
**0.10%**	18,256 ± 347^b^	3,052 ± 70^b^	2,840 ± 81^b^	221 ± 3^b^	17,771 ± 302^b^	13.19 ± 0.06^b^	0.26 ± 0.03
**0.50%**	10,277 ± 267^c^	1,145 ± 91^c^	2,168 ± 159^c^	219 ± 6^b^	15,676 ± 416^c^	12.95 ± 0.11^b^	—
**1%**	6,976 ± 147^d^	784 ± 69^d^	1,849 ± 61^d^	213 ± 4^b^	14,573 ± 265^d^	12.49 ± 0.07^c^	—
**2%**	2,994 ± 117^e^	376 ± 38^e^	965 ± 84^e^	209 ± 6^b^	13,669 ± 399^e^	12.17 ± 0.09^d^	—

Note: Standard deviation is represented as ±SE (n = 3); columns with different letters indicate a significant difference at the *P* ≤ 0.05 level on the basis of Duncan’s multiple range test.

## Discussion

Tobacco is well-known for its metal accumulating capabilities; several species have been investigated over the years because Cd accumulation in leaves is known to be higher than in the stem and root^16, 21^. It is also clear that the metal concentration in tobacco varies along the stalk, higher in older leaves than in younger top leaves^21^. Early work demonstrated that Cd accumulates at highest levels in the oldest tobacco leaves^16^. These results are consistent with time-dependent deposition of Cd in leaves; in other words, Cd accumulation increases with leaf age and is likely a permanent mechanism^16^. The results of our pot and field experiments show that the concentrations of Cd in spring growth and regenerated tobacco occur in the order, lower leaves > middle leaves > upper leaves > root > stalk (or > stalk > root). Such a difference in Cd concentrations in various plant parts is not unique to tobacco, this characteristic is seen in many plant species^22^. However, the strong ability of tobacco leaves to accumulate Cd may be due to the absorption of this metal in contaminated soil by roots and subsequent movement through the conductive system^23^. Vogeli-Lange and Wagner^24^ have suggested that Cd can be rapidly transported from root to shoot, probably within a few hours, a finding that is in agreement with the results of Rosén *et al*.^25^ who noted that the relatively high concentrations of this metal in tobacco leaves are reflected in a high TFp (ratio: shoot Cd/root Cd). Thus, tobacco meets an important condition as a hyperaccumlator, according to the criteria outlined by Brooks^26^.

It is well-known that BCF values, proxies for the ability of plants to uptake metals, also depend on soil characteristics^27^. However, although the phytoremediation of Cd contaminated soil using tobacco has been the subject of some preliminary research, this plant has not previously been considered a hyperaccumulator^19, 28^. Normal concentrations of Cd in tobacco leaves grown in unpolluted soils range between 1 mg kg^−1^ and 3 mg kg^−1^ ^29^. However, a high BCF of Cd in tobacco leaves has been reported not only under highly contaminated soil conditions but also under lower conditions of exposure. Cai *et al*.^30^, for example, found BCF values higher than 10 in tobacco leaves grown in Dayu county, Jiangxi Province, China, where the concentration of Cd in soil was only about 1 mg kg^−1^. In this study, highest BCF values were found in lower leaves while values for field and pot experiments were 37.53 (second cutting) and 41.11 (first cutting) for the Chaling soil, and 19.21 (second cutting) and 23.45 (first cutting) for the Guanxi soil (Table 2). Results show that BCF values decreased with increasing soil Cd concentration, consistent with previous results^31^. Thus, our results for tobacco are consistent with another condition of hyperaccumulators^26^, and this plant could be considered for the remediation of Cd contaminated soil, as recently suggested in other studies^20^.

Numerous factors, including tobacco cultivars, soil characteristics, agronomic practices, and environment conditions, affect Cd uptake by tobacco. Of these, it is thought that soil characteristics (e.g., pH and the concentration of bioavailable Cd) are most important in accounting for the accumulation of this metal in tobacco^32, 33^. Indeed, pH has a negative influence on available Cd concentration in soil^34^; Cd adsorption by clay irons, manganese oxides, and organic matter decreases in concert with pH^35^. The two soil types considered in this study were both red and acid, with pHs of 4.8 and 5.4, respectively. The concentrations of total Cd in these soils were 0.59 mg kg^−1^ and 1.35 mg kg^−1^, respectively, while available concentrations were 0.42 mg kg^−1^ and 0.66 mg kg^−1^, respectively (Table 1).The significantly high Cd concentration in tobacco leaves under pot and field conditions reported here (Fig. 1 and Table 1) could thus be due to high availability in the two low pH soils. Our results show that tobacco Cd concentrations are highly positively correlated with the content of this metal in soil^20^, consistent with several earlier studies on other plants^36, 37^. However, Cd uptake and translocation may differ substantially among species and cultivars of tobacco^18, 38^. In one comprehensive study conducted between 2001 and 2003, 755 samples collected from13 countries in tobacco-producing regions showed that Cd concentrations ranged between 0 mg kg^−1^ and 6.78 mg kg^−1^. The tobacco cultivar K326 used in this study was one of the highest in the sample^33^.

Levels of Cd in field situations are typically low, usually less than 1 mg kg^−1^, and just a fraction of this will be absorbed by plants^39^. As a result, the concentration of Cd in tobacco leaves under field conditions will be lower than that seen in pot or hydroponic experiments. Results in this study show that Cd concentrations in pot experiment tobacco leaves after the first cut were higher than in the field experiment, while concentrations in lower and middle tobacco leaves in the field after the second cut increased to a level slightly higher than the pot experiment, which decreased after the second cut. This clear difference between natural field and controlled pot conditions may be due to different physiological conditions of both the plant and soil environment^40, 41^. Total Cd in soil is also controlled and limited in the pot experiment, which will affect accumulation of Cd in regenerated leaves. In contrast, abiotic conditions are variable and more realistic in the field; regeneration of tobacco under field conditions not only does not influence the potential of leaves to accumulate Cd, but also improves uptake because the ripening time of regenerated tobacco is longer than that for first growth plants.

Two approaches were used to determine phytoextraction efficiency: (1) Cd removal efficiency from the top soil (0–20 cm) expressed in terms of the decline percentage of overall soil concentration^42^; and (2) Cd removal efficiency from top soil in a theoretical case, calculated as % of soil metal removed by one crop = (plant metal concentration × biomass)/(soil metal concentration × soil mass in the rooting zone) × 100^43, 44^. For comparative purposes, these two methods were adopted in this study to evaluate the phytoextraction efficiency of Cd from soil by regenerated tobacco. Results show that both theoretical and actual phytoextraction efficiencies of Cd by tobacco after two cuts in pot experiments are significantly higher than in field experiments. This is because just 15 kg of soil requires remediation by a potted tobacco plant, while almost eight times as much soil in a field will require remediation. In addition, total Cd extraction efficiency in practice (i.e., 9.43% for Chaling soil and 6.24% for Guanxi soil) under field conditions is confirmed by theory (10.0% for Chaling, 6.73% for Guanxi), indicating that the remediation potential of Cd by regenerated tobacco is significant. Thus, regenerated tobacco plants will be more efficient at removing Cd from light or moderately contaminated acidic soils.

Although successful phytoextraction generates large volumes of contaminated plant biomass, very few studies have addressed the issue of disposal. One approach, the use of liquid extraction to remove heavy metals from harvested biomass has been described in several studies^45–47^, and HCl is one of the most commonly applied reagents. This approach works efficiently because metal extraction from soils or solids is more effective under acidic conditions^48^. Our leaching experiments demonstrate that just a 0.5% solution of HCl can remove 98.4% of Cd from tobacco leaves, a removal efficiency that is not significantly different compared to 2% HCl following three successive extractions. Subsequent concentrations of Cd in tobacco leaves were lower than permissible levels established by the Hygienic Standard for Feeds in China (i.e., ≤0.5 mg kg^−1^; GB13078-1991; Fig. 3). In addition, nicotine was not detected in residual leaves following all treatments with 70% ethanol, and although the concentrations of nutrient elements (i.e., Ca, Mg, P, and K) decreased significantly following HCl extraction, a lower reduction was seen in crude protein and Fe levels compared to residual leaves after extraction.

Leaf proteins are extremely abundant and have a high nutritional value^49^. Thus, these have been considered as an alternative protein source that might remediate the problem of a soaring world population coupled with limited cultivable land and a general food shortage^49–51^. Proteins from leaves can be used in food, animal feed, or hydrolyzed to amino acid for other applications^51–55^. Tobacco leaf residue is one example of a potential protein source; the F1 protein is most abundant in tobacco, can be obtained from leaves via a relatively simple procedure in a pure, tasteless form, and has an essential ammo acid composition similar to egg or milk proteins^56^. Less is known, however, about the economics underlying the utilization of tobacco as a protein source. On the basis of prices for other plant protein feeds, such as soybean and oil cake, expected income for a farm would be between US$260 and US$500 per ton of tobacco leaves. Although it is not as lucrative to grow tobacco for protein as it is for smoking; local farmers may accept this revenue stream as they can gain more income than for other remediation plants during the clean-up period.

In summary, the findings of our study suggest that tobacco after being cut twice still appears to accumulate high Cd in leaves in moderately contaminated acidic soils. Compared to other crops, regenerated tobacco produces a large amount of biomass and enhances the removal efficiency of Cd from soil. We have also shown that the concentration of Cd in tobacco can meet the limits for feeds in China following three successive extractions with 0.5% HCl. At the same time, these extractions have little influence on the protein content of leaf residues, which means that they could provide a rich source of plant protein for food. This technique proposed here for the phytoremediation of Cd contaminated soil makes effective use of waste farmland in both time and space, could create considerable revenues for local farmers, and efforts to bring Cd concentration down to an acceptable level (<0.3 mg kg^−1^) in soil according to China Standard (GB15618-1995). In addition, this approach presents both an effective treatment and novel utilization for tobacco products.

## Materials and Methods

### Materials and preparation

This experiment was conducted at two distinct sites that comprise agricultural soils contaminated with low levels of Cd, one in Chaling in Zhuzhou County, and one in Guanxi in Chenzhou County. According to soil characteristics presented in Table 5, acidic red soils (pH 4.8 and pH 5.4, respectively) are present at both sites; the concentration of total Cd was 0.587 mg kg^−1^ in Chaling and 1.347 mg kg^−1^ in Guanxi, while available Cd concentrations were 0.423 mg kg^−1^ and 0.663 mg kg^−1^, respectively. The tobacco cultivar used in all experiments was K326, seeds of which were provided by the Zhuzhou Tobacco Monopolistic Company, Hunan Province, China. Seeds were generated and grown in a culture medium in a greenhouse, and tobacco seedlings were transplanted to a pot or the field for experiments after generation.

**Table 5:** Soil properties.

Soil properties	Location	
	Chaling	Guanxi
pH (soil:H_2_O = 1:5)	4.70	5.40
CEC (cmol kg^−1^)	12.76	16.32
Organic matter (g kg^−1^)	33.62	24.37
Total N (g kg^−1^)	2.19	1.12
Total P (mg kg^−1^)	685.13	408.75
Total Cd (mg kg^−1^)	0.59	1.38
Available Cd (mg kg^−1^)	0.42	0.66

### Pot experiments

All the pot experiments reported here were carried out in a greenhouse at Hunan Agricultural University, Hunan, China. Polluted soils were sampled at the Chaling and Guanxi sites, were loaded into pots (i.e., 31 cm× 28 cm; 15 kg of soil per pot), and each soil treatment was replicated six times. One tobacco seedling was transplanted into each pot; fertilization and cultivation management were the same as in field experiment (N 150 kg ha^−1^, P 150 kg ha^−1^, and K 375 kg ha^−1^). At first cut (after 60 days), three pots each were allocated at random for harvesting of whole tobacco plantlets, while plants in another three pots were cut at two-thirds stalk length, fertilized again. After 80 days, the regenerated whole plants along with the top 20 cm of soil were harvested. The concentration of Cd in all parts of the tobacco plant and soil were determined after two successive cuts, and the dry weights of leaves and stalks were recorded.

### Field experiments

The field experiments at the two sites comprised three plots each 10 m ×10 m planted with tobacco. The plots were fertilized with N (150 kg ha^−1^), P (150 kg ha^−1^), and K (375 kg ha^−1^) in order to provide the necessary nutrients for plant growth before tobacco seedlings were transplanted to the field at a density of 2.25 seedlings per m^2^. The first cut was taken after 60 days; mature tobacco plants were cut at two-thirds of stalk length so as to allow them to regenerate into complete plants. At the same time, three randomly selected tobacco samples (including roots, stalks, and leaves) were harvested from each plot. Following the first cut, plots were re-fertilized and allowed to re-grow without interruption until 80 days when three further samples (including roots, stalk, and leaves) as well as the top 20 cm of soils were harvested from each plot. The concentration of Cd in all parts of the tobacco plant and soil were determined after two successive cuts, and the dry weights of leaves and stalks were recorded.

### Removal of Cd from tobacco leaves

Tobacco leaves from both pot and field experiments were dried, ground, and mixed. Two factors, including different concentrations (i.e., 0.1%, 0.5%, 1.0%, and 2.0%) of HCl in 70% ethanol solutions, and three successive extraction procedures were chosen to evaluate the effect of HCl on the removal of Cd from tobacco leaves. Batch extraction experiments were conducted in 60 ml centrifuge bottles that contained 1.00 g of powdered leaves alongside 20 ml of different concentrations of HCl (1:20 *w*/*v*). These bottles were agitated on a rotary shaking table at a speed of 40 r min^−1^ for 12 hours at room temperature. After extraction, the sample was de-watered in a centrifuge at 4,000 rpm for 10 minutes and rinsed three times with 30 ml of deionized water. Leaves remaining in the bottle were drained and dried at 60°C for 72 hours, and all experiments were conducted in triplicate. Concentrations of Cd in tobacco leaves after each extraction, as well as nicotine and nutrients (crude protein, Ca, Mg, Fe, K, and P), after three successive extractions were analyzed.

### Sampling and analysis

Soil samples were collected from the top 20 cm of the field surface, air dried, and homogenized in an agitate mortar to pass through a 1mm sieve. Total soil Cd was determined following mixed acid (HCL-HNO_3_-HCLO_4_) digestion^57^, while analysis of available soil Cd concentration was determined via extraction with 0.1 mol L^−1^ KCl. Soil pH (soil/H_2_O = 1:5, v/v) was determined using a pH meter (PHS-3C).

Tobacco samples, including roots, stalks, upper leaves (eight pieces), middle leaves (nine pieces), and lower leaves (eight pieces), were rinsed with tap water before being washed with deionized water. All plant samples were then oven dried at 105°C for two hours and then at 60°C until completely dry (48 hours). Subsequently, dried plant samples were then ground into a powder using a knife mill (WB200, Wei Bo Chuang, Beijing), and this was digested in a mixture of HNO_3_/HClO_4_ (85:15%, *v*/*v*) to determine total Cd and nutrient elements (Ca, Mg, Fe, K, and P)^58^. The concentrations of Cd, Ca, Mg, Fe, K, and P in all samples were determined using either Optima 8300 ICP-OES (PerkinElmer, USA) or graphite furnace flame atomic absorption spectrometry in a SpectrAA-GTA120 (Varian, USA). Crude protein contents were determined via Kjeldahl analysis with a factor of 6.25^59^, while determination of nicotine in tobacco leaves follows the method reported by Saunders and Blume^60^.

### Statistical analysis

BCF was calculated using Eq. 1, as follows:

1 $${\rm{B}}{\rm{C}}{\rm{F}}=\frac{{C}_{{\rm{C}}{\rm{d}}-{\rm{a}}{\rm{b}}{\rm{o}}{\rm{v}}{\rm{e}}{\rm{g}}{\rm{r}}{\rm{o}}{\rm{u}}{\rm{n}}{\rm{d}}/{\rm{r}}{\rm{o}}{\rm{o}}{\rm{t}}}}{{C}_{{\rm{C}}{\rm{d}}-{\rm{s}}{\rm{o}}{\rm{i}}{\rm{l}}}}$$

In this expression, *C*_Cd-aboveground_ (mg kg^−1^) is the Cd concentration measured in the aboveground parts of the plant, including the stalks and leaves, while *C*_Cd-soil_ (mg kg^−1^) is the Cd concentration in the soil.

Theoretical total Cd uptake was calculated using Eq. 2, as follows:

Field experiment:

2 $$\begin{array}{rcl}{\rm{Metal}}\,\mathrm{uptake}\,\,({\rm{g}}\,{{\rm{ha}}}^{-1}) & = & {{\rm{C}}}_{{\rm{metal}}{\rm{concentration}}{\rm{in}}{\rm{plant}}{\rm{tissue}}}({\rm{mg}}\,{{\rm{kg}}}^{-1})\\  &  & \times {{\rm{W}}}_{{\rm{plant}}{\rm{dry}}\mathrm{weight}}({\rm{kg}}\,{{\rm{m}}}^{-2})\times {{\rm{S}}}_{{\rm{cultivated}}{\rm{area}}}({\rm{ha}})\end{array}$$

Pot experiment:

3 $$\begin{array}{rcl}{\rm{Metal}}\,{\rm{uptake}}\,({\rm{g}}\,{{\rm{pot}}}^{-2}) & = & {{\rm{C}}}_{{\rm{metal}}{\rm{concentration}}{\rm{in}}{\rm{plant}}{\rm{tissue}}}({\rm{mg}}\,{{\rm{kg}}}^{-1})\\  &  & \times {{\rm{W}}}_{{\rm{plant}}{\rm{dry}}\mathrm{weight}}({\rm{kg}}\,{{\rm{plant}}}^{-1})\times {{\rm{n}}}_{{\rm{number}}{\rm{of}}{\rm{plants}}}\end{array}$$

Theoretical phytoextraction efficiency (%) of harvested tobacco was calculated using Eq. 3 ^41^, as follows:

Field experiment:

4 $$\begin{array}{c}{\rm{Phytoextraction}}\,{\rm{efficiency}}\,( \% )\\ \quad \quad \quad =\,\frac{{{\rm{C}}}_{{\rm{Cd}}{\rm{in}}{\rm{plant}}{\rm{tissue}}}({\mathrm{mg}\mathrm{kg}}^{-})\times {{\rm{W}}}_{{\rm{plant}}{\rm{dry}}\mathrm{weight}}({\rm{kg}}\,{{\rm{m}}}^{-2})\times {{\rm{S}}}_{{\rm{cultivated}}{\rm{area}}}\,({\rm{ha}})}{{{\rm{C}}}_{{\rm{Cd}}{\rm{in}}\mathrm{soil}}\times 1.3\,{\rm{t}}\,{{\rm{m}}}^{-3}\times 20{\rm{cm}}\times {{\rm{S}}}_{{\rm{cultivated}}{\rm{area}}}({\rm{ha}})}\times 100 \% \end{array}$$

Pot experiment:

5 $$\begin{array}{c}{\rm{Phytoextraction}}\,{\rm{efficiency}}\,( \% )\\ \quad \quad \quad =\,\frac{{{\rm{C}}}_{{\rm{Cd}}{\rm{in}}{\rm{plant}}{\rm{tissue}}}({\rm{mg}}\,{{\rm{kg}}}^{-})\times {{\rm{W}}}_{{\rm{plant}}{\rm{dry}}\mathrm{weight}}({\rm{kg}}\,{{\rm{pot}}}^{-1})\times {{\rm{n}}}_{{\rm{number}}{\rm{of}}{\rm{plants}}}}{{{\rm{C}}}_{\mathrm{Cd}{\rm{in}}\mathrm{soil}}\times 15\,{\rm{kg}}\,{{\rm{pot}}}^{-1}\,}\times 100 \% \end{array}$$

The phytoextraction efficiency (%) of the top soil (0–20 cm) in practice was calculated using Eq. 4, as follows:

6 $${\rm{Phytoextraction}}\,{\rm{efficiency}}\,( \% )=\frac{{C}_{\mathrm{Cd}-{\rm{soil}}}({\rm{mg}}\,{{\rm{kg}}}^{-})-{c}_{{\rm{Cd}}-{\rm{soil}}{\rm{after}}\mathrm{remediation}}({\rm{mg}}\,{{\rm{kg}}}^{-})}{{C}_{\mathrm{Cd}-{\rm{soil}}}({\rm{mg}}\,{{\rm{kg}}}^{-})}\times 100 \% $$

All dates are presented as means and standard deviations using Origin 8.0 and Microsoft Excel software. One-way ANOVAs and Duncan’s multiple range tests (*p* ≤ 0.05) calculated using the statistical package SPSS V21were applied to compare the differences in heavy metal concentrations in crops, oils, and seed meals, as well as total metal uptake.
